# Tau pathology does not affect experience-driven single-neuron and network-wide *Arc/Arg3.1* responses

**DOI:** 10.1186/2051-5960-2-63

**Published:** 2014-06-10

**Authors:** Nikita Rudinskiy, Jonathan M Hawkes, Susanne Wegmann, Kishore V Kuchibhotla, Alona Muzikansky, Rebecca A Betensky, Tara L Spires-Jones, Bradley T Hyman

**Affiliations:** Alzheimer’s Disease Research Laboratory, Department of Neurology, MassGeneral Institute for Neurodegenerative Disease, Massachusetts General Hospital, Harvard Medical School, Charlestown, 02129 MA USA; Novartis Institutes for Biomedical Research, Inc., Cambridge, 02139 MA USA; Brigham and Women’s Hospital, Boston, 02115 MA USA; New York University School of Medicine, Skirball Institute of Biomolecular Medicine, New York, 10016 NY USA; Biostatistics Center, Massachusetts General Hospital, Boston, 02114 MA USA; Department of Biostatistics, Harvard School of Public Health, Boston, 02115 MA USA; Centre for Cognitive and Neural Systems and the Euan MacDonald Centre for Motorneurone Disease Research, University of Edinburgh, Edinburgh, EH8 9JZ UK

**Keywords:** Alzheimer’s disease, Tau, Neurofibrillary tangles, Arc, Neuronal activity, Activity-dependent expression

## Abstract

Intraneuronal neurofibrillary tangles (NFTs) – a characteristic pathological feature of Alzheimer’s and several other neurodegenerative diseases – are considered a major target for drug development. Tangle load correlates well with the severity of cognitive symptoms and mouse models of tauopathy are behaviorally impaired. However, there is little evidence that NFTs directly impact physiological properties of host neurons. Here we used a transgenic mouse model of tauopathy to study how advanced tau pathology in different brain regions affects activity-driven expression of immediate-early gene Arc required for experience-dependent consolidation of long-term memories. We demonstrate in vivo that visual cortex neurons with tangles are as likely to express comparable amounts of *Arc* in response to structured visual stimulation as their neighbors without tangles. Probability of experience-dependent Arc response was not affected by tau tangles in both visual cortex and hippocampal pyramidal neurons as determined postmortem. Moreover, whole brain analysis showed that network-wide activity-driven Arc expression was not affected by tau pathology in any of the brain regions, including brain areas with the highest tangle load. Our findings suggest that intraneuronal NFTs do not affect signaling cascades leading to experience-dependent gene expression required for long-term synaptic plasticity.

## Introduction

Intraneuronal neurofibrillary tangles (NFT) composed of misfolded hyperphosphorylated tau proteins are one of two stereotypical types of lesions present in the Alzheimer’s brain (together with amyloid plaques) [1]. The extent of neurofibrillary pathology correlates well with synaptic loss, neuronal loss, glial activation, and cognitive decline [2] and tangles have long been considered a primary therapeutic target in Alzheimer’s disease (AD). Moreover, mutations in tau have been linked to neurodegeneration in several tauopathies, including FTDP-17 [3], and cerebral tau aggregates are present in many disorders [4].

Despite the compelling proof for a role of misfolded tau in neurodegenerative disease, little data directly test the hypothesis that tangles, per se, impair neuronal function. In fact, there is no conclusive evidence for a mechanistic role of NFTs in dysregulation of nervous system, either on a single-neuron or neuronal network level. We hypothesized that the tangle pathology would disrupt integration of incoming information in individual neurons with NFTs and that the brain networks most affected by tangles would have impaired neuroplastic properties when responding to sensory inputs in vivo. As a proxy of experience-dependent network integration and plasticity we used the activity-dependent transcription of a memory-related immediate-early gene *Arc*. Activity-driven expression of *Arc* is crucial for synaptic tagging and remodeling in response to sensory and behavioral inputs (reviewed in [5–9]) and is often used as a reporter of expression of neuroplasticity in excitatory neurons.

We quantitatively assessed the impact of tangle pathology on the experience-driven *Arc* responses after a behaviorally relevant, well characterized visual stimulus paradigm [10–13] to determine whether there are cell-specific or network-wide plasticity deficits directly linked to NFTs. We crossed the rTg4510 mice which express P301L mutant form of human tau and develop advanced tangle pathology [14], with a previously characterized fluorescent reporter line of *Arc* transcription [10, 15]. Using intravital fluorescent brain microscopy we found that the presence of NFTs in visual cortex neurons did not affect the amplitude of Arc responses to the stimulation. Postmortem odds ratio analysis revealed that the probability of Arc response in individual neurons in both visual cortex and hippocampus is not affected by expression of mutant tau and/or presence of tau tangles. Quantitative analysis of all brain regions with detectable neuronal Arc expression after visual stimulation showed no differences in characteristics of network-wide Arc responses between control and mutant mice, even in the brain areas with the highest tangle load. Finally, reduction of brain-wide soluble human tau concentration by suppression of mutant tau expression in the rTg4510 mice did not affect Arc responses. These results indicate that behavioral and physiological deficits observed in mice expressing P301L mutant of human tau are not mediated by alterations of post-synaptic pathways involved in activity-dependent expression of immediate-early genes such as Arc.

## Results

### Tau pathology does not affect the amplitude of experience-driven *Arc*responses in the visual cortex

**Figure 1:**
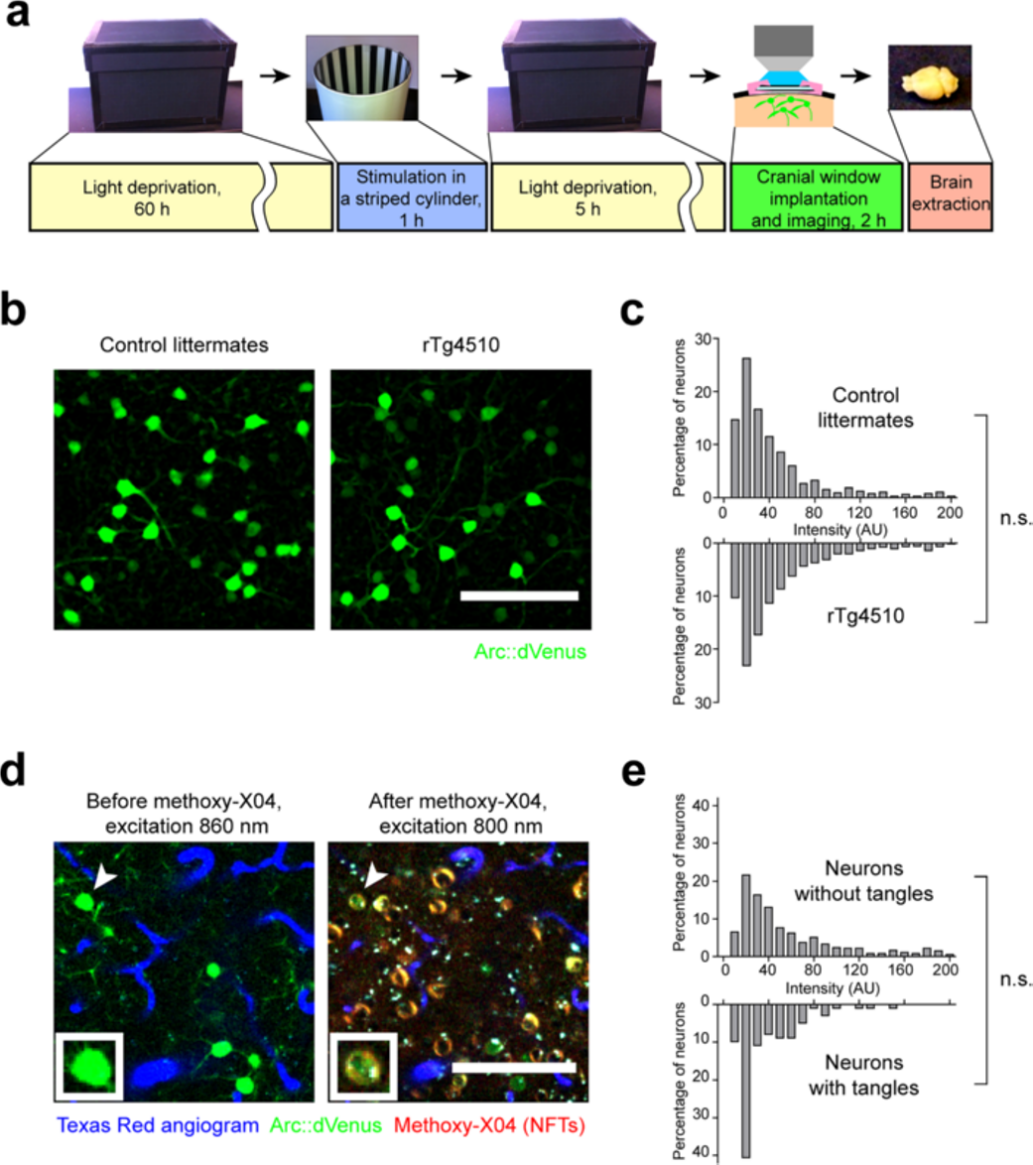
**In vivo quantification of Arc::dVenus reporter in the visual cortex of rTg4510 mice. (a)** Experiment outline. Structural visual stimulation paradigm described previously in [10] was followed by cranial window implantation over the medial extrastriate visual cortex and imaging on a multiphoton microscope. After the imaging, the brains were collected and processed for immunohistochemical analysis. **(b)** Sample maximum intensity projections of in vivo-acquired multiphoton z-stacks from the layer II/III of the medial extrastriate visual cortex, showing Arc::dVenus reporter (shown in green) expression induced by the visual stimulation. Scale bar = 100 μm. **(c)** Frequency distribution histograms of activity-induced Arc::dVenus fluorescence in individual visual cortex neurons of rTg4510 mice (n = 8 mice, 2212 neurons) and control littermates (n = 5 mice, 1043 neurons). AU, arbitrary units. n.s., not significant, P = 0.27. **(d)** Sample single-section in vivo multichannel micrographs of the same visual cortex site from an rTg4510 mouse before (left) and after (right) the intravenous delivery of a tangle-binding dye methoxy-X04 (red). Fluorescent angiogram (blue) was used as a landmark pattern during the image analysis. Arrowheads points to a dVenus-positive neuron with an NFT and the inset shows a close-up view of this neuron. Scale bar = 100 μm. Inset size = 20 × 20 μm. **(e)** Frequency distribution histograms of activity-induced Arc::dVenus fluorescence in rTg4510 neurons without tangles (n = 4 mice, 720 neurons) and with tangles (n = 4 mice, 101 neurons). AU, arbitrary units. n.s., not significant, P = 0.083.

To study activity-induced *Arc* induction in vivo in the brain of rTg4510 mice, we used *Arc::dVenus* reporter and visual stimulation experimental paradigm similar to our previous set of experiments with *Arc::dVenus* × *APP/PS1* strain [10]. The well characterized *Arc::dVenus* reporter line expresses destabilized bright yellow fluorescent protein, dVenus, under the control of the *Arc* promoter and allows quantification of activity-driven transcriptional response of *Arc* gene in both living mice and postmortem brain tissue [10, 15]. Triple transgenic *Arc::dVenus × CamKII::rtTA × tetO::Tau(P301L)* (*Arc::dVenus × rTg4510*) mice of tangle-bearing age (11–12 months old) and littermate *Arc::dVenus × CamKII::rtTA* controls were housed in light-proof dark enclosures for 60 hours prior to being exposed for 1 hour to structured visual stimulation in a glass cylinder with alternating black and white stripes illuminated from the outside (Figure 1a). This type of visual stimulation induces robust expression of Arc::dVenus in, among other brain areas, the anteromedial aspect of extrastriate visual cortex reaching a maximum in roughly 6 hours (Figure 1b, also see [10]). After the stimulation, the mice were returned to their home cages and placed into the dark enclosures for 5 hours. At the end of the second light deprivation period mice were anesthetized, implanted with a cranial window over the right visual cortex and imaged with a 2-photon microscope (Figure 1a). First, the mice were imaged using 860 nm excitation laser to allow optimal simultaneous detection of dVenus signal (Figure 1b) and Texas Red-conjugated dextrans which were injected intravenously to create a reference fluorescent angiogram. Image segmentation and quantification of dVenus signal in individual neurons showed no difference in dVenus expression level distributions between rTg4510 mice and littermate controls (P = 0.27, Figure 1c) and the shape of the histograms was similar to the data from *Arc::dVenus* controls in [10]. After dVenus imaging, the blood–brain barrier-permeable Congo Red derivative dye methoxy-X04 [16] mixed with Texas Red dextrans was injected intravenously. Methoxy-X04 has previously been shown to efficiently label NFTs in post-mortem brain tissue from human AD subjects [16] and transgenic mice expressing mutant tau [17]. After a short incubation to allow drug diffusion in the brain, the same visual cortex area was re-imaged with 800 nm excitation to visualize methoxy-X04-labeled NFTs and the angiogram (Figure 1d). Precise overlay of dVenus and methoxy-X04 images of the same cortical areas allowed us to determine whether individual dVenus-positive neurons had tangles or not. We found no significant difference in dVenus expression levels, representing the amplitude of *Arc* response, between tangle-free and tangle-bearing neurons (P = 0.083, Figure 1e).

### NFTs do not change the probability of *Arc*response in the host neurons

**Figure 2:**
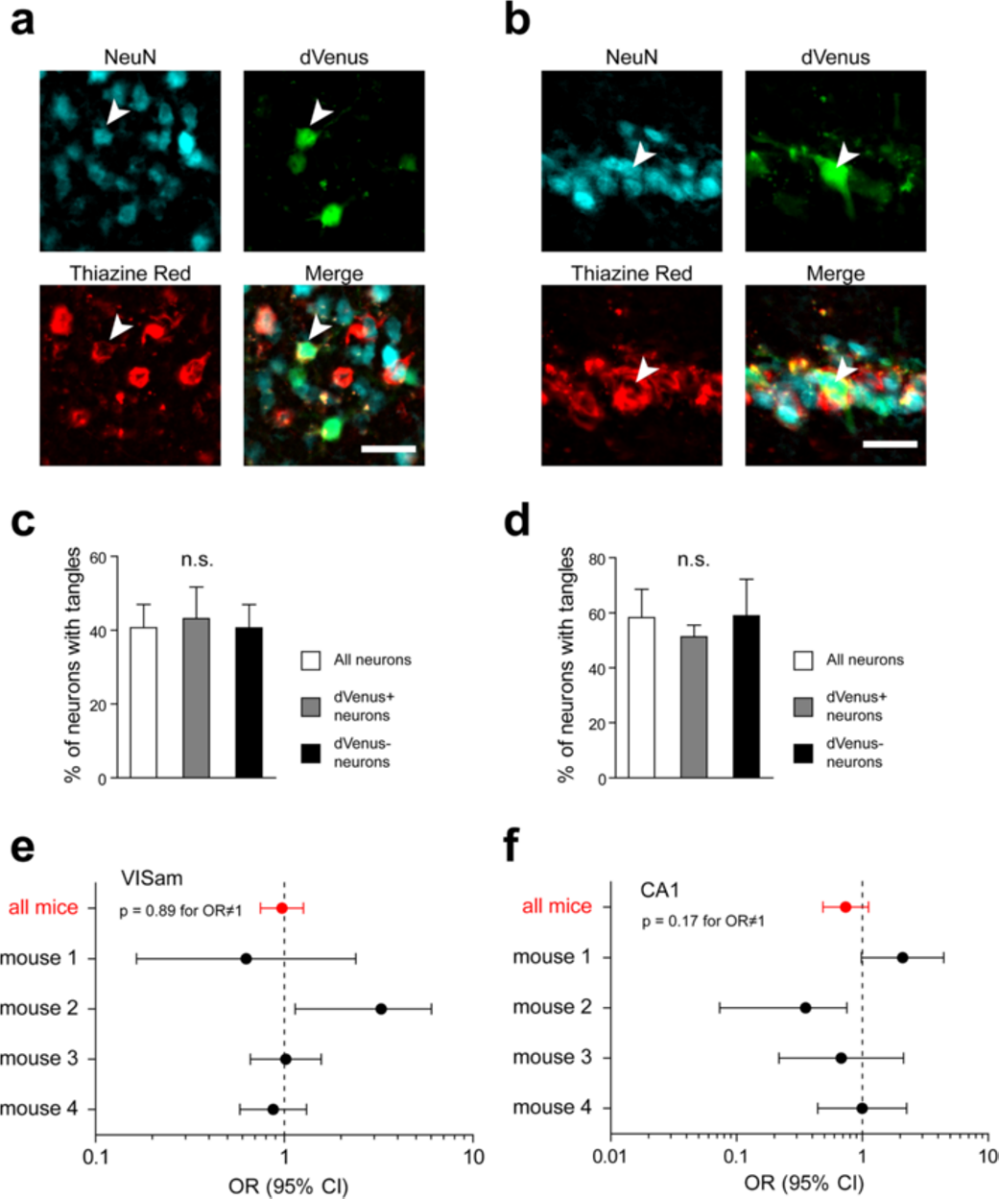
**Postmortem analysis of Arc response probability in cortical and hippocampal neurons with and without NFTs. (a, b)** Immunohistochemical staining of medial extrastriate visual cortex **(a)** and CA1 field of the hippocampus (b) of an rTg4510 mouse. NeuN immunolabeling is shown in cyan, direct dVenus fluorescence in green and thiazine red labeling of tangles in red. Scale bar = 25 μm. Arrowheads in a and b point to Arc::dVenus-positive neurons with tangles. **(c, d)** Stereological estimations of the percentage of neurons with tangles based on counts of NeuN-, dVenus- and thiazine red-positive neurons in the medial extrastriate visual cortex **(c)** and CA1 **(d)**. Data presented as means ± s.e.m.; n.s., not significant, P = 0.96 in c, P = 0.84 in d. N = 4 mice, 4273 neurons (visual cortex) and 441 neurons (CA1). (e,f) Forest plot of odds ratios (ORs) of NFTs affecting neuronal Arc responses in extrastriate medial visual cortex **(e)** and CA1 field of the hippocampus **(f)**. Presented data were acquired from the analysis of stereological counting of neurons using Fisher’s exact test and shown as OR ± 95% confidence interval on a log10 scale.

Having found that tangles do not influence the amplitude of *Arc* responses in the active neurons, we next asked whether they might affect the overall probability of response. Following in vivo multiphoton imaging, mice were sacrificed and their brains were processed for postmortem immunohistochemical analyses. We produced coronal sections of the entire forebrain and first analyzed a subset of these sections spanning anteromedial extrastriate visual cortex (VISam) and CA1 field of the hippocampus (at the level of approx. -2.5 mm from Bregma along the rostro-caudal axis according to [18]). These regions were selected for in-depth analysis because the former was the principal visual area exhibiting *Arc* response to this type of stimulation [10] and was examined in vivo. The latter is known to upregulate *Arc* following exposure to a novel environment [19, 20] and was shown to have deficits in *Arc* mRNA expression in the rTg4510 mouse model [21]. The sections were immunolabelled with anti-NeuN antibodies and treated with a red-spectrum fluorescent dye, thiazine red, which has high affinity for beta-pleated fibrillar structures and labels NFTs [22, 23]. dVenus fluorescence could be observed directly (Figure 2a,b). Using stereological quantification we found no difference in percentage of neurons with NFTs between all NeuN-labeled neurons, dVenus-positive and dVenus-negative neurons in either layer II/III of VISam (P = 0.96, Figure 2c) or pyramidal layer of CA1 (P = 0.84, Figure 2d). In accord with the lack of difference in stereological counts of dVenus positive responsive neurons with tangles, statistical analysis of odds ratio for NFTs affecting probability of Arc::dVenus expression in individual host neurons was not significantly different from 1 (VISam: P = 0.89, Figure 2e; CA1: P = 0.17, Figure 2f).

### Tau pathology does not affect brain-wide *Arc*responses

**Figure 3:**
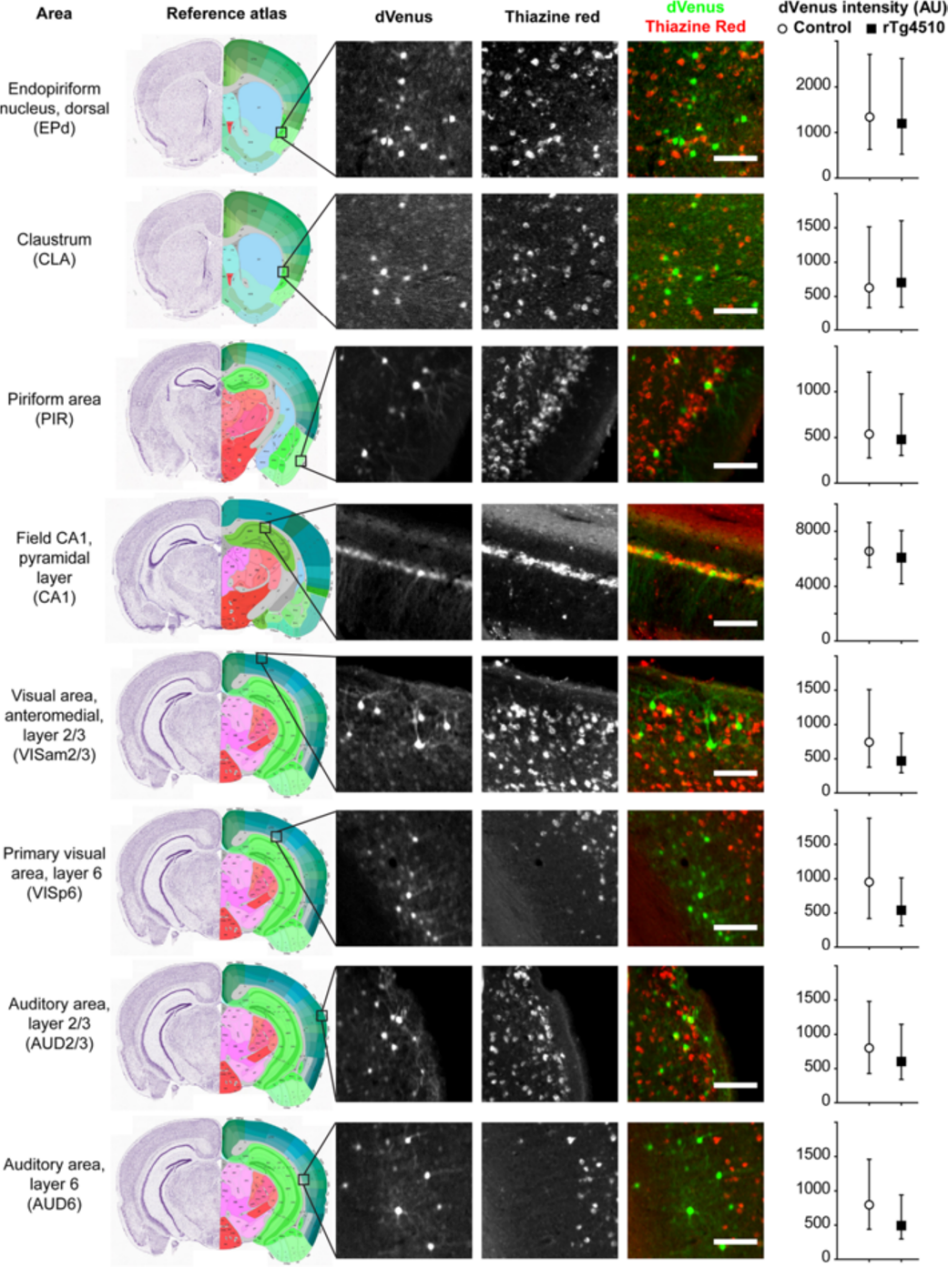
**Whole-forebrain histochemical analysis of Arc::dVenus expression.** Experience-dependent dVenus expression in various forebrain regions following structured visual stimulation and NFT labeling (Thiazine Red fluorescence) in the brains of rTg4510 mice . Scale bar = 100 μm. dVenus intensities in individual neurons are presented as medians with interquartile ranges. N = 4 control littermates, n = 5 transgenic mice. P-values for Arc::dVenus expression comparison between rTg4510 mice and control littermates: EPd, P = 0.80 (control: n = 293 neurons; transgenic: 419); CLA, P = 0.77 (control: n = 291 neurons; transgenic: 427); PIR, P = 0.57 (control: n = 776 neurons; transgenic; 604); CA1, P = 0.92 (control: n = 394 neurons; transgenic: 396); VISam2/3, P = 0.08 (control: n = 1298 neurons; transgenic: 519); VISp6, P = 0.18 (control: n = 952 neurons; transgenic: 974); AUD2/3, P = 0.65 (control: n = 1427 neurons; transgenic: 1057); AUD6, P = 0.05 (control: n = 739 neurons; transgenic: 962)). Reference atlas image credit: Allen Institute for Brain Science, Allen Mouse Brain Atlas.

*Arc* can be expressed in excitatory neurons in various neuronal circuits in response to relevant sensory and behavioral inputs [24–26]. In order to assess the effects of tau pathology on *Arc* expression at the network-wide level we analyzed the sets of coronal sections spanning the entire forebrain (from 2 mm to −4.5 mm from Bregma along the rostro-caudal axis according to [18]) with 500 μm interval. Sections were labeled with thiazine red to visualize NFTs and DAPI, and imaged on a three-channel setup, including a yellow filter set to detect direct dVenus fluorescence. We identified 8 distinct areas that had a significant number of the Arc::dVenus-positive neurons in all examined mice, both transgenic and control littermates (Figure 3). Among these areas were visual (VISp VI, VISam II/III, auditory (AUD IV and II/III) and piriform (PIR II, III) cortices, pyramidal layer of CA1 field of the hippocampus and two frontal subcortical association areas: claustrum (CLA) and dorsal endopiriform nucleus (EPd). Certain regions, such as motor, somatosensory, anterior cingulate, entorhinal, posterior parietal cortices and dentate gyrus of hippocampus had Arc::dVenus-positive neurons in some, but not all, analyzed brains, without correlation to genotype (data not shown), indicating that the response in these areas was not specific to this type of behavioral stimulation. We found no areas with significant response in control littermates that had a complete absence of response in the same region in transgenic mice or vice versa. None of the eight above-mentioned areas had any significant differences in the levels of Arc::dVenus between transgenic mice and control littermates (Figure 3).

**Figure 4:**
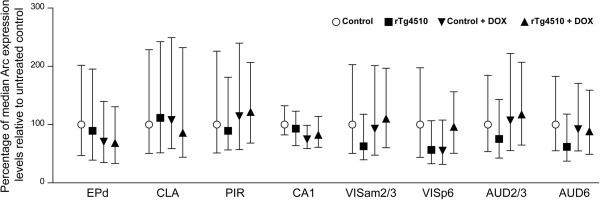
**Effects of suppression of mutant tau expression on Arc::dVenus responses.** Arc::dVenus expression in different brain regions of rTg4510 mice and control littermates is presented as medians with interquartile ranges. No significant effect of doxycycline treatment on Arc::dVenus responses were observed in either transgenic or control mice, when corrected for multiple testings. Numbers of mice and neurons for untreated control littermate and transgenic mice are as indicated in Figure 3 legend. Numbers of mice and neurons for doxycycline-treated group are as following: n = 5 control littermates, 3 transgenic mice; EPd: control: n = 449 neurons, transgenic: 267; CLA: control: n = 480 neurons, transgenic: 272; PIR: control: n = 1091 neurons, transgenic: 527; CA1: control: n = 479 neurons, transgenic: 196; VISam2/3: control: n = 1060 neurons, transgenic: 729; VISp6: control: n = 730 neurons, transgenic: 534; AUD2/3: control: n = 1394 neurons, transgenic: 1098; AUD6: control: n = 697 neurons, transgenic: 736.

Previous studies have shown that the expression of soluble mutant tau is largely responsible for behavioral and physiological deficits and neurotoxicity in the rTg4510 model [14, 27]. We took advantage of the fact that the transgene expression in the rTg4510 mice can be suppressed by doxycyclin treatment [14, 21, 27] to determine whether removal of soluble mutant tau in aged mice affects brain-wide Arc responses. A cohort of 11–12 months old transgenic and control littermate mice received doxycycline-supplemented diet for 6 week, after which they underwent visual stimulation as described above and their brains were processed for the brain-wide Arc::dVenus expression analysis. We found no effect of doxycycline treatment on activity-driven Arc responses in either control or transgenic mice in none of brain regions with consistent detectable Arc expression (Figure 4).

**Figure 5:**
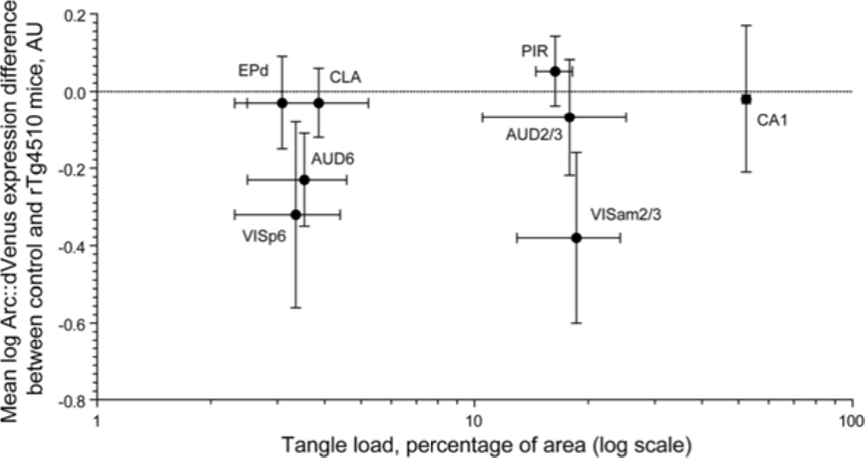
**NFT load does not correlate with the differences in Arc expression between rTg4510 and control mice.** Surface area occupied by tangles (presented as means ± s.e.m. on log10 scale for clarity) in the forebrain regions with significant Arc expression (presented in Figure 3) in rTg4510 mice plotted versus mean (±s.e.m.) log Arc::dVenus expression difference between control and rTg4510 mice. Spearman R = 0.13, P = 0.75. N = 4 control littermates, 4 transgenic mice.

As an additional measure of interaction between the NFT pathology and experience-driven Arc expression, we examined whether quantified tangle load in different brain regions correlated with differences in Arc expression levels between rTg4510 mice and control littermates. In fact, we found no significant correlation between NFT load expressed as percentage of brain area section occupied by tangles and experience-driven differential Arc expression expressed as mean log Arc expression difference between transgenic mice and control littermates (Figure 5).

## Discussion

This study aimed to elucidate the effects of tau pathology on experience-dependent neuroplasticity in individual neurons and local neuronal networks. We applied an experimental paradigm that we have recently developed to address a similar question in the context of Aβ pathology [10]. Reporter mice expressing a fluorescent protein under the control of the plasticity-related immediate-early gene *Arc* promoter [15] were crossed to a model of tauopathy based on P301L mutation in the human tau protein [14]. We studied experience-induced *Arc* reporter expression in the brains of these mice with a combination of in vivo longitudinal multiphoton imaging and postmortem immunohistochemical analysis. Using quantitative in vivo measurements of *Arc* transcription reporter followed by detection of NFTs in large volumes of the visual cortex of a living mouse brain, we showed that tangles did not affect the absolute *Arc* expression levels in the individual responses of neurons. At the same time, neurons with NFTs were as likely to have *Arc* responses as neurons without NFTs, as determined postmortem in the same brain area. Similar to visual cortex neurons, CA1 pyramidals with NFTs had the same likelihood of Arc responses as their neighbors without tangles. Taking into account the complex postsynaptic signaling and transcriptional machinery required for the activity-driven expression of the *Arc* gene [7, 8, 28–30], this demonstrates, for the first time, that despite being large cytoplasmic space-occupying inclusions, NFTs do not significantly disrupt the integration of synaptic inputs and the downstream execution of memory-related genetic programs. The data also do not demonstrate any changes in Arc responses to a sensory stimulus due to the soluble mutant tau ubiquitously expressed in the CamKII-positive neurons in this mouse model. There is a possibility that NFTs or tau indeed introduce changes to Arc responses small enough that they could not be detected with our imaging and analysis methods. However, in our previous study using this quantification technique with similar cohort sizes (and thousands of neurons examined) [10] we were able to resolve significant changes as small as 15% of median expression level postmortem and 25% in vivo. It is unlikely that if smaller undetected changes were induced by NFTs, they would have significant effect on neuronal circuit function, as these changes would be orders of magnitude lower than Arc responses to physiological stimuli [10, 11]. These conclusions are in accord with a recent study from our laboratory showing intact function of tangle-bearing and tangle-free neurons in the visual cortex of rTg4510 mice as demonstrated by in vivo multiphoton imaging of visual activity-evoked calcium transients [31].

Our findings of unchanged *Arc* transcription in the visual cortex contrasted with a previous study from our laboratory showing decreased *Arc* expression in the CA1 pyramidal layer of the hippocampus in the rTg4510 model mice after exploration of a novel environment [21] and with the earlier mRNA profiling of the human AD brains which found lower amounts of the *Arc* mRNA in tangle-bearing CA1 neurons compared to tangle-free neurons [32]. To gain a “brain-wide” perspective on the tau pathology effect on neuronal networks, we processed entire brains of rTg4510 mice crossed with *Arc* reporter strain to identify anatomical regions with detectable *Arc* expression following the exposure of the mice to the structured visual stimulation. We found no regions that lacked activation in one genotype and were active in the other and vice versa, which indicates that the widespread tau pathology does not fundamentally change brain wiring patterns. We also detected no overt differences in experience-dependent network-wide Arc expression levels between transgenic mice and control littermates in any of the examined brain regions. Even the brain areas with the highest tangle load (such as CA1 field of the hippocampus where more than 50% of the brain section was occupied by tangles) exhibited normal Arc responses to the visual stimulation.

We considered the differences in experimental paradigm between the initial study of hippocampal responses to exploration of a novel environment [21] and the current study, which focused on visual cortical responses to defined visual stimuli. Since the retina and lateral geniculate are disease free in this model, the latter stimulus was designed to specifically probe somatodendritic responses and integration of signals in which the axons carrying the primary input are intact, while the behavioral/memory aspects of the exploratory behavior task requires integration of widely dispersed information in the cortex and concurrent signaling to the hippocampus for further processing from areas that are markedly impacted by both NFT and neuronal loss. It may be that this latter form of integrative function is more dependent on the integrity of axonal processes and on the relative timing and intensity of feedforward and feedback signals, and so Arc reporting of signal in the hippocampus is inherently more sensitive to subtle effects compared to visual cortical responses. Nonetheless, the agreement between the studies that the presence of NFT does not impact the likelihood of an Arc response, despite the substantially different behavioral paradigms and the method of assessing Arc (in situ hybridization v.s. Arc promoter-driven fluorescent reporter) emphasizes that the type of defect in signal integration caused by a tangle, if any, is quite subtle.

Overall, our findings show that NFTs do not significantly alter postsynaptic function of the host neurons which is required for the experience-dependent Arc expression. Given the importance of meticulous *Arc* regulation for the proper functioning of synaptic machinery [33–36] and the role that activity-dependent *Arc* expression plays in memory-related physiology through AMPAR endocytosis [35], structural plasticity [37] and synaptic tagging [38], our data suggest that mechanisms not linked to experience-dependent immediate early genes are behind behavioral phenotypes in rTg4510 mice and cognitive impairment in AD. A number of studies have demonstrated electrophysiological, functional and morphological changes in neurons from rTg4510 mice at the advanced stage of tau pathology [28, 39–42], and, notably, increased intrinsic excitability in the cortical neurons, with evidence indicating that these changes could not be attributed to the presence or absence of NFTs in the affected neurons [40, 42]. Taken together with our data, NFTs appear not to cause major disruptions of synaptic integration of information.

## Materials and methods

### Mice

All animal experimentation was performed in conformance with institutional and US National Institutes of Health guidelines and approved by the Massachusetts General Hospital Institutional Animal Care and Use Committee. *Arc::dVenus* reporter mice expressing destabilized variant of a bright yellow fluorescent protein dVenus under 7.1 kb mouse *Arc* promoter [15] were crossed with suppressible *CamKII::rtTA × tetO::Tau (P301L)* (rTg4510) strain [14]. *Arc::dVenus × CamKII::rtTA* non-carriers of *Tau (P301L)* transgene were used as littermate controls to account for potential effects of *rtTA* transactivator overexpression. Genotyping was performed using polymerase chain reaction (PCR) with three pairs of primers targeting *dVenus*, *rtTA* and *Tau (P301L)*. For mutant tau suppression experiment, a cohort of mice (n = 5 control, n = 3 transgenic) was fed chow containing 200 mg/kg doxycycline (Harlan Teklad) for 6 weeks before visual stimulation. All mice used in this study were 11–13 months old.

### Visual stimulation, surgery and in vivo multiphoton imaging

Structured visual stimulation was performed as described previously [10]. Briefly, single-housed mice were placed in their home cages into the dark light-proof ventilated enclosures for 60 hours prior to beginning of stimulation to suppress visual experience-induced *Arc::dVenus* expression. After the end of light deprivation mice were transferred to an illuminated glass cylinder with alternating vertical black and white stripes for 1 hour. Following the end of the visual stimulation, mice were placed back into the home cages and put into light deprivation chambers for 5 hours before the anesthesia for cranial window implantation was induced with 4% isoflurane in balanced oxygen inside of the dark light-proof enclosure for 5 minutes and maintained at the level of 1.2–1.6% during the surgery and consequent imaging while keeping the body temperature at 37°C. Cranial windows were implanted over the right visual cortex as described previously [10, 43]. Immediately after cranial window implantation Texas Red-conjugated dextran (MW 70,000 Da, 12.5 mg/mL in sterile PBS, Molecular Probes) was injected IV to provide fluorescent angiogram used as landmark pattern for co-registration of two consecutive imaging sessions and as a reference fluorophore to monitor cranial window clarity and appearance of bleedings. Mice were imaged on multiphoton Olympus Fluoview 1000 MPE system equipped with mode-locked titanium/sapphire MaiTai laser (Spectra-Physics) and Olympus BX61WI upright microscope with XLPLN 25× water-immersion objective (NA = 1.05). Z-stacks in both imaging sessions were acquired with the resolution of 1 μm/pixel in X-Y dimension with the Z-step of 3 μm. For each mouse the imaged site consisted of 2×4 512×512×240 μm stacks acquired with 10% overlap in X–Y dimensions resulting in an imaged field in visual cortex spanning roughly 1×2×0.24 mm^3^ ((m–l) × (r–c) × (d–v)). For the first imaging session, excitation laser was tuned to 860 nm with the output power set to 76 mW before the objective. Emitted light was collected in three channels: 460–500 nm (autofluorescence), 530–560 nm (dVenus fluorescence) and 575–630 nm (Texas Red dextran angiogram). Then, methoxy-X04 (5 mg/kg) [44] diluted in Texas Red dextran solution (12.5 mg/mL in sterile PBS) was injected IV to label NFTs. 30 minutes after methoxy-X04 injection, a second imaging of the same site was performed with the excitation laser tuned to 800 nm for more efficient detection of metoxy-X04 fluorescence, with 100 mW output power before the objective. In this session, the 460–500 nm channel collected emitted light from methoxy-X04 bound to the tangles and other two channels contained the same dVenus and Texas Red dextran signals as in the first sessions and were used for consequent co-registration of the two image stacks. Imaging settings were kept constant between mice and the laser output power was calibrated before each imaging session using infrared photometer.

### Processing and quantification of in vivo imaging data

Only image stacks acquired during the first imaging session (860 nm excitation, before methoxy-X04 injection) were used for quantification of Arc::dVenus expression levels to avoid potential spectral crosstalk with methoxy-X04. Arc::dVenus expression levels were processed using Fiji package of NIH ImageJ software (fiji.sc; rsbweb.nih.gov/ij) as described previously [10]. Both stacks (before and after methoxy-X04 injection) were aligned side-by-side based on dVenus and Texas Red dextran patterns (as on Figure 1d) and each quantified dVenus-positive neuron was manually assigned a tangle-positive or tangle-negative status.

### Post-mortem tissue analysis

After the end of in vivo imaging still anesthetized mice were transcardially perfused with ice-cold phosphate-buffered saline (PBS) followed by 4% paraformaldehyde in PBS. Brains were incubated in fixative at 4°C for 48 hours and 50 μm free-floating sections were cut on a Microm HM400 microtome. Coronal sections were consecutively collected into 10 tubes, so each tube contained a set of sections covering the full forebrain with 500 μm interval. For the cell counting, appropriate sections were first incubated in thiazine red (MP Biomedicals, 0.01% in PBS) for 20 min to label NTFs and washed 3 times with PBS, followed by immunolabeling with mouse anti-NeuN antibody (1:500, Cat. No MAB377, Millipore) and secondary goat-anti-mouse AlexaFluor 350 (1:500, Cat. No A11045, Molecular Probes). Stereological counting of *Arc::dVenus*- and NeuN-positive neurons was performed on Olympus CAST system in neuronal layer II/III of medial extrastriate visual cortex and pyramidal layer of CA1 field of the hippocampus. Images for Figure 2 were acquired on Zeiss Axio Observer. Z1 fluorescent microscope equipped with 40× objective. For the full-brain analysis, an entire set of sections spaced at 500 μm from each mouse was labeled with thiazine red mixed with DAPI (0.2 μg/ml), washed for 1 hour in 1% Triton-X100 in PBS and 3 times in PBS. Full-section tile scan was performed on Zeiss Axio Observer. Z1 microscope with 5× objective run by MetaMorph (Molecular Devices) using following three filter sets: Ex/Em 365/445 nm (DAPI), 500/545 nm (direct dVenus fluorescence) and 545/620 (thiazine red). Stitched multichannel images were manually aligned to Allen Mouse Brain Reference atlas (brain-map.org). All image processing was performed in Fiji package of ImageJ. To quantify Arc::dVenus in individual cells, regions of interest with Arc::dVenus-positive neurons in the dVenus channel were cropped to save computation time, background subtracted, images thresholded and segmented with “Analyze particles” plug-in to identify cell bodies. The cell body masks were then applied to the pre-thresholded images to measure mean fluorescence intensity in each identified cell body. To quantify NFT load, brain regions of interest were outlined based on reference atlas alignment and DAPI labeling. Crop masks were applied to the thiazine red channel, images thresholded and percentage of area occupied by tangles calculated.

### Statistical analysis

The normality of all datasets was tested using Kolmogorov-Smirnov method. Non-normal datasets of *Arc::dVenus* expression levels in individual neurons with variable sample sizes (Figure 1 and 3) were compared using Wilcoxon rank-sum test with correction for clustering of values within individual mice [45]. Comparison of normal data with identical sample sizes (Figure 2c,d) was performed using one-way ANOVA with Bonferroni post-test. The associations between tangles and *Arc* responses and corresponding odds ratios (Figure 2) were analyzed using 2-way contingency table analysis with Fisher’s exact test (GraphPad Prism 5); this assumes independence across neurons. To assess the effects of doxycycline treatment (Figure 4) we used a mixed effects model to adjust for treatment and genotype and their interaction, with adjustment for correlation within mouse. Each region was modeled separately. We corrected for multiple testing across the eight brain regions using a Bonferroni correction. Calculation of Spearman R and correlation P-value for normal tangle load data with identical sample sizes (Figure 5) was performed on average values across mice.
